# Ribbon Synapse Plasticity in the Cochleae of Guinea Pigs after Noise-Induced Silent Damage

**DOI:** 10.1371/journal.pone.0081566

**Published:** 2013-12-09

**Authors:** Lijuan Shi, Lijie Liu, Tingting He, Xiaojing Guo, Zhiping Yu, Shankai Yin, Jian Wang

**Affiliations:** 1 Department of Physiology and Pharmacology, Medical College of Southeast University, Nanjing, China; 2 School of Human Communication Disorders, Dalhousie University, Halifax, Canada; 3 Department of Otolaryngology, 6^th^ Affiliated Hospital, Jiaotong University, Shanghai, China; National Institutes of Health/NICHD, United States of America

## Abstract

Noise exposure at low levels or low doses can damage hair cell afferent ribbon synapses without causing permanent threshold shifts. In contrast to reports in the mouse cochleae, initial damage to ribbon synapses in the cochleae of guinea pigs is largely repairable. In the present study, we further investigated the repair process in ribbon synapses in guinea pigs after similar noise exposure. In the control samples, a small portion of afferent synapses lacked synaptic ribbons, suggesting the co-existence of conventional no-ribbon and ribbon synapses. The loss and recovery of hair cell ribbons and post-synaptic densities (PSDs) occurred in parallel, but the recovery was not complete, resulting in a permanent loss of less than 10% synapses. During the repair process, ribbons were temporally separated from the PSDs. A plastic interaction between ribbons and postsynaptic terminals may be involved in the reestablishment of synaptic contact between ribbons and PSDs, as shown by location changes in both structures. Synapse repair was associated with a breakdown in temporal processing, as reflected by poorer responses in the compound action potential (CAP) of auditory nerves to time-stress signals. Thus, deterioration in temporal processing originated from the cochlea. This deterioration developed with the recovery in hearing threshold and ribbon synapse counts, suggesting that the repaired synapses had deficits in temporal processing.

## Introduction

Noise exposure at relatively low levels or doses has been found to cause “silent” damage to the afferent cochlear innervation [1]–[3]. It has been called “silent” damage because the noise exposure does not cause a permanent threshold shift in hearing, which is currently the major criterion for noise-induced hearing loss and noise safety standards.

In both mice and guinea pigs, noise exposure can cause massive damage in the ribbon synapses between inner hair cells (IHC) and type I spiral ganglion neurons (SGN) [1]–[3]. In mice, this damage is largely irreparable [1], [3], resulting in large-scale degenerative SGN death that developed slowly after the initial damage [1].

In guinea pigs, however, initial damage of a similar degree was found to be largely repairable, as indicated by the recovery in ribbon counts [2]. Correspondingly, much less long-term SGN death was found than in mice [3]. Furthermore, the temporal processing resolution of the auditory system, as tested with paired click-evoked auditory brainstem responses (ABRs), deteriorated in guinea pigs within a month after noise exposure, while the hearing threshold recovered fully during this period [2]. These results suggest a clear cross-species difference in the repair process around the ribbon synapses, and that the repaired synapses are not fully functionally intact.

The afferent synapses between IHCs and SGNs are mainly of the typical ribbon type [4]–[9]. The ribbon structure has been considered to facilitate the release and recycling of neurotransmitters, and is thus responsible for both quick responses to rapidly changing signals and long-lasting responses to continuous stimuli. Ribbon synapses are recognized to play an important role in cochlear temporal processing [4]–[6], [10].

Thus, it is understandable that massive damage to the ribbon synapses could compromise their temporal resolving power. However, it is unclear presently whether the deteriorated ABR to time-stress stimuli originates from the cochlea and how it is related to the repair process of the ribbon synapses.

In this study, we further explored the effects of noise exposure on the temporal processing ability of the auditory system. Instead of observing the ABR, we focused on cochlear responses by measuring the compound action potential (CAP) to time-stress signals. We also observed the damage and the repair of ribbon synapses at both pre- and post-synaptic sites in an attempt to provide insight into the plastic changes to the ribbon synapses during the repair process.

## Methods

### Animals and general experimental protocol

Male albino adult (2–3 months old) guinea pigs were obtained from Qinglongshan Animal Farm, Jiangning, Nanjing, China. All animals used passed Preyer reflex testing, an otoscopic exam, and showed normal hearing, as determined with tone burst-evoked ABR. Their body weights were 300–350 g when recruited. All animal procedures were approved by the University Committee for Laboratory Animals of Southeast University, China (Permit number: SYXK 2011-0009).

In total, 40 guinea pigs were used; they were divided into the control (*n* = 10) and experimental (*n* = 30) groups. A baseline ABR test was performed in all animals. Then, the 30 animals in the experimental group were exposed to a broadband noise at 105 dB SPL for 2 h. The ABR test was repeated in 10 of the 30 guinea pigs in this group 1 day, 1 week, and 1 month post-noise exposure (1 DPN, 1 WPN, and 1 MPN). At each of the three time points, the 10 guinea pigs were sacrificed for morphological examination after the CAP test. The 10 animals in the control group were tested for ABR and CAP and then sacrificed for morphology at the same time as the 10 subjects in the noise group tested at 1 MPN. At each time point, observations of ribbon synapses were performed successfully in 6–10 cochleae. The norm for ribbon synapses was established successfully in six cochleae from six animals in the control group.

### Noise exposure

The animals in the noise group were exposed to a single dose of broadband noise at 105 dB SPL for 2 h. During the exposure, the animals were awake and unrestrained in a cage 60 cm below the horns of two loudspeakers; one was a low frequency woofer and the other was a high frequency tweeter. Electrical Gaussian noise was delivered to the speakers after power amplification. The acoustic spectrum of the sound was distributed mainly between 1 and 20 kHz as reported previously [2]. The noise level was monitored using a ¼-inch microphone linked to a sound level meter (Larson Davis 824, USA).

### Physiological tests

For ABR and CAP recordings, the animal was anesthetized with ketamine + xylazine (40 mg/kg+10 mg/kg, respectively, i.p.) and the body temperature maintained at 38°C with a thermostatic heating pad. Three subdermal needle electrodes were used to record ABRs. To record CAPs, a silver ball electrode was placed on the round window membrane via an opening in the mastoid. The electrode was fixed in place with dental cement. The reference and grounding electrodes were subdermal needles inserted behind the ears. TDT hardware and software (BioSig and SigGen) were used for stimulus generation and bio-signal acquisition. The acoustic stimuli used were: 1) tone bursts of 10-ms duration with cos^2^ gating, 0.5-ms rise/fall time, and 2) equal-level paired clicks of 80 µs with inter-click intervals (ICI) varying from 20 to 0.5 ms. The stimuli were played through a speaker (MF1, TDT), placed 10 cm in front of the animal's ears The evoked responses were sampled at 25 kHz and pre-amplified with a TDT RA16PA with a gain of 20 and averaged 1000 times for ABR and 100 times for CAP. For measuring hearing threshold, the ABR was recorded with tone bursts presented at a rate of 21.1/s at frequencies from 1 to 32 kHz in octave steps. At each frequency, the test was performed in a down sequence, starting from 90 dB SPL and in 5-dB steps until the ABR response disappeared. The threshold was determined as the lowest level at which a repeatable wave III response could be obtained. To evaluate auditory temporal processing ability, CAP was recorded in response to paired clicks with an overall repetition rate of 11.1/s. The clicks were presented at three suprathreshold levels (60, 70, and 80 dB peSPL). The amplitude of CAP to the second click (CAP2) was measured as a function of ICIs to show the response change to time stress.

### Morphology

After the end-point functional tests, the cochleae in the experimental group were harvested. One ear from each animal was used for immunostaining against ribbons and PSDs, and the other ear was for hair cell counts. Because we found no hair cell loss, those data are not reported. To examine the ribbon synapse structure, the cochlea was quickly placed in cold PBS, and then perfused rapidly with 4% paraformaldehyde in PBS buffer followed by a brief post-fixation at 4°C for 1 h. The cochlea was then transferred back into PBS and the bone over the middle ear-facing portion of the cochlear spiral was removed with fine forceps. After removing the tectorial membrane, the cochlea was permeabilized with 1% Triton X-100 in PBS for 60 min, incubated for 60 min in 5% goat serum in PBS and then incubated in the mixture of two primary antibodies (mouse anti-CtBP2 (C-terminal-binding protein 2) IgG1 from BD Biosciences, cat. # 612044, 1∶200) and mouse anti-PSD95 IgG2a from Millipore, cat. # MAB1596, 1∶1000) overnight at 4°C. This was followed by treatment with secondary antibodies (goat anti mouse IgG1 and IgG2, 1∶1000, Invitrogen A21124 and A21131 respectively) for 2 h at room temperature. All antibodies used were diluted in 5% goat serum in PBS. After immunostaining, the cochlea was then decalcified in 5% EDTA and the basilar membrane was dissected into five or six pieces, mounted on microscope slides and coverslipped. To reduce the variability and increase the reliability of results, control and experimental samples from the same time points were processed together under the same conditions.

Confocal images were acquired using a confocal laser-scanning microscope (Zeiss LSM 510 META) with ×100 oil-immersion objectives. Image stacks were then ported to image-processing software (Lsmix and ImageJ). The laser excitation power and microscope emission and detection settings were kept the same across the different observations. Across the whole basilar membrane, immunoreactive puncta of CtBP2 and PSD95 were counted across a total of 10 frequency regions, from 1 to 40 kHz (see Figures). The locations in terms of lengths or distances from the apex were mapped according to the previously published norm for guinea pig cochleae [11]. In each region, the counting was done from all IHCs that were seen in 2–3 microscopic fields, each typically having 9–11 IHCs. The total puncta were divided by the total number of IHC nuclei to obtain the averaged number of ribbons and PSDs for each IHC. The areas of ribbon and PSD puncta were defined by the number of voxels having intensity values higher than a pre-selected threshold; the same criterion was used for all samples.

To determine the distance between ribbons and IHC nuclei or PSD and IHC nuclei, the central locations of the region of interest (including ribbons, PSDs, and IHC nuclei) were calculated as the center of mass of the fluorescent spots. This was defined in ImageJ as the brightness-weighted average of the *x* and *y* coordinates of all pixels in the region of interest. However, the distances between ribbons/PSDs and the IHC nuclei were calculated using only the value of the *x* coordinate of the center, which is in the longitude direction of IHCs.

### Statistical analysis

All data are expressed as means ±SEM and *post hoc* multiple comparisons were performed using Tukey tests following ANOVAs. The SigmaStat for Windows software was used for these statistical analyses.

## Results

### A. Damage to and repair of afferent innervation to IHCs

**Figure 1:**
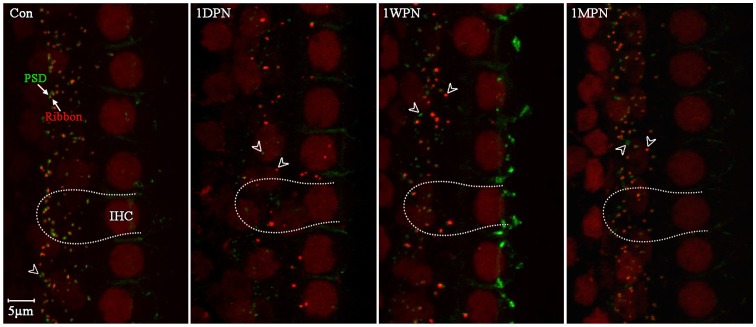
Confocal images of ribbons (stained red against CtBP2) and PSD95 (green) showing the noise-induced changes in numbers, sizes, and locations. 1: 1 day, 1 week, and 1 month post-noise. The short hollow arrows point to ribbons or PSDs that were not paired.

Frequency-specific ABRs showed a clear threshold elevation across the whole frequency range tested at 1 DPN and a total recovery at 1 WPN, as reported previously [2]. In this study, post-synaptic terminals were stained with an anti-PSD95 antibody. The signal was concentrated at the terminal but was also seen in the membrane of IHCs. The signal was strong at and above the reticular laminar, but weak at the lower surface and did not interfere with the observation of much stronger and highly concentrated, rounded signals on the postsynaptic membrane. Figure 1 shows typical changes in ribbons and PSDs at different times after the noise exposure, compared with that of the control. The images in this figure were taken from the cochlear region around 20–22 kHz. In the control cochlea, both the ribbons and PSDs were mostly located at the bottom of the IHCs and paired with each other, although a small proportion of PSDs was found not to be paired with ribbons (indicated by short hollow arrows in Fig. 1). At 1 DPN, the numbers of ribbons and PSDs were both greatly reduced. Because ribbon loss was higher than PSD loss, many PSDs stood alone without ribbons (short hollow arrows in Fig. 1). Moreover, many ribbons were dislocated towards the IHC nuclei. From 1 week to 1 month after noise exposure, both ribbon and PSD counts largely, but not completely, recovered. This was also true for their location. However, the numbers of ribbons and PSDs at 1 MPN were still lower than in the control, and the distributions of the ribbon and PSD punctas were higher than that of the control.

**Figure 2:**
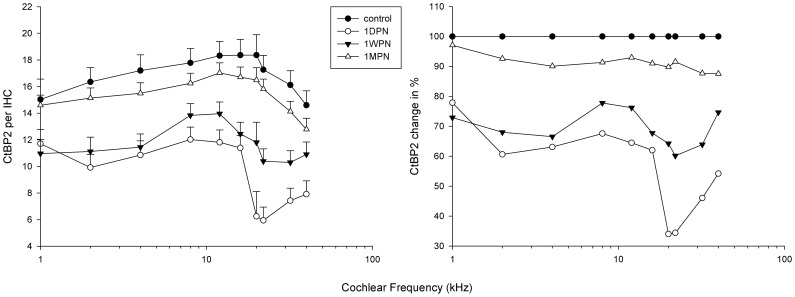
Noise-induced changes in the frequency distribution of ribbon counts along the cochleae. Left: absolute values of ribbon counts. Right: normalized percentage (control values as 100%). 1 DPN, 1 WPN, and 1 MPN: 1 day, 1 week, and 1 month post-noise.

**Figure 3:**
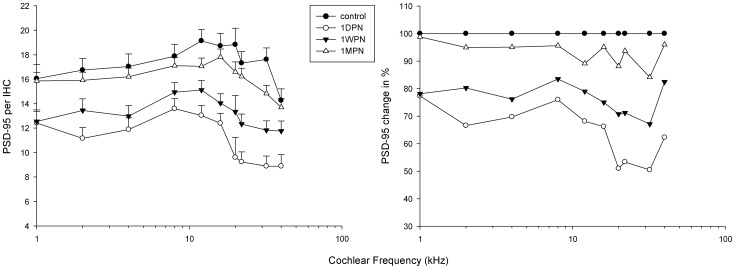
Noise-induced changes in the frequency distribution of PSD counts along the cochlea. Left: absolute values of ribbon counts. Right: normalized percentage (control values as 100%). 1 DPN, 1 WPN, and 1 MPN: 1 day, 1 week, and 1 month post-noise.

Figures 2 and 3 show the number of CtBP2/PSD per IHC as a function of cochlear frequency position in guinea pigs using a previously established tonotopic map [11]. The data are presented as the number of ribbons/PSDs per IHC on the left and percentage against the control values in both figures. An overall loss of 41.7% ribbon count was seen at 1 DPN. This was reduced to 30.3% and 8.8% at 1 WPN and 1 MPN, respectively. The initial loss of PSDs was 35.0% at 1 DPN and was reduced to 23.3% and 6.8% at 1 WPN and 1 MPN, respectively.

**Figure 4:**
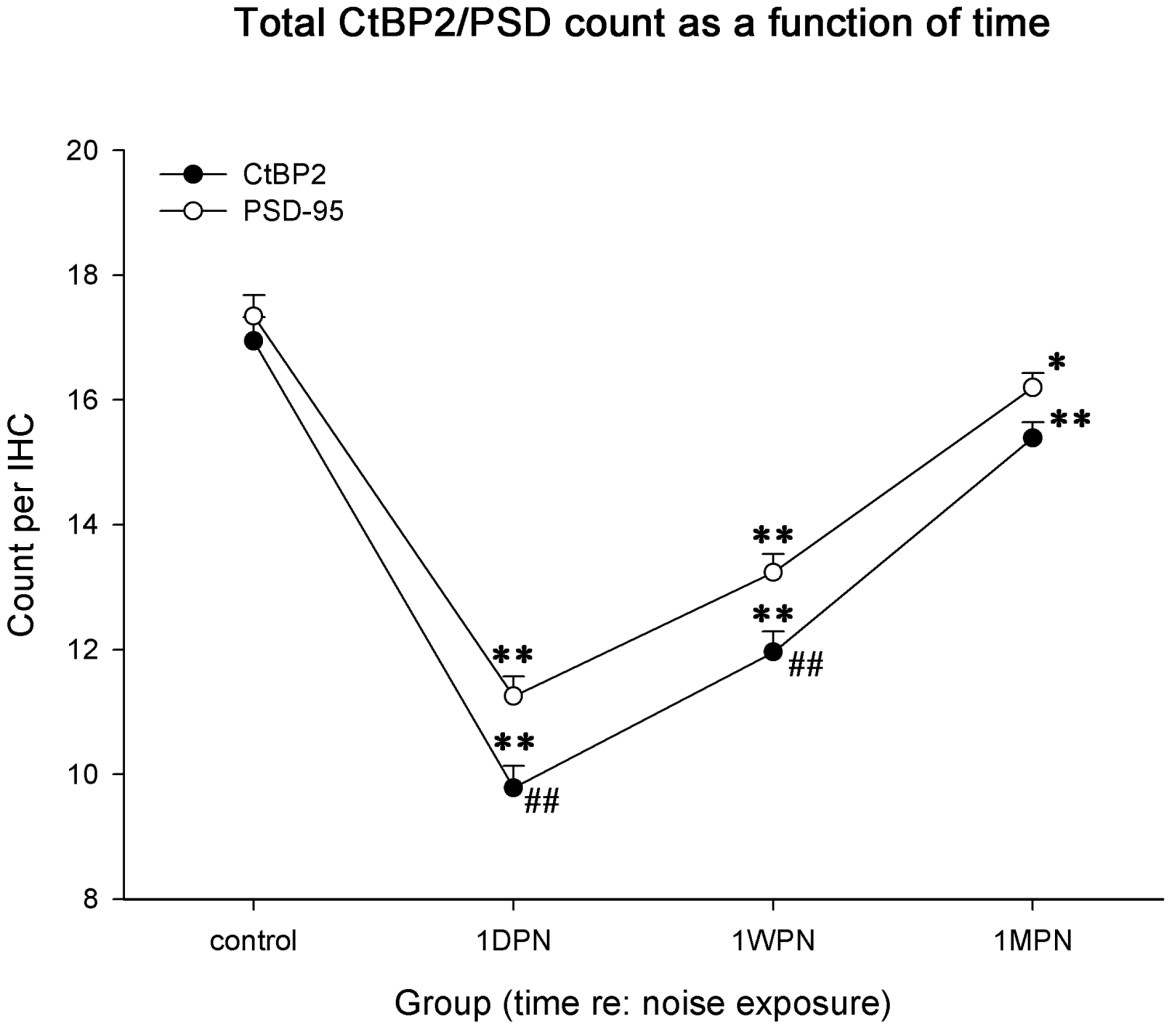
Changes in averaged PSD/ribbon counts as a function of time versus noise. A significant decrease in both ribbon and PSD counts was seen after the noise. The counts had not fully recovered at 1* comparison with the PSD and ribbon controls, respectively. # comparison between ribbons and PSDs.

Figure 4 shows the changes in averaged PSD and ribbon counts per IHC as a function of post-noise time. A two-way ANOVA was performed against time and counted targets (PSD vs. ribbon). A significant effect of time was found for both PSD and ribbon counts (*F_3,79_* = 164.1, p<0.001). *Post hoc* tests showed that at each time point after the noise, both PSD and ribbon counts were significantly lower than that of the corresponding controls (indicated by *s in Fig. 4). A significant effect of the targeted synaptic components was also seen (*F_1,79_* = 19.0, p<0.001): the loss of PSDs was significantly lower than the loss of ribbons at 1 DPN and 1 WPN (#s in Fig. 4).

**Figure 5:**
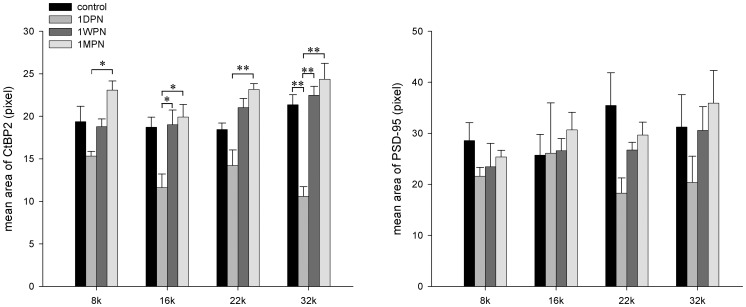
Noise-induced changes in sizes of ribbons and PSDs over four frequency spots.

In addition to the number changes, the ribbon size and location were also changed by the noise exposure. Figure 5 shows the changes in the average size of both PSDs and ribbons at four frequency regions after noise exposure. The average ribbon size was reduced after noise exposure. This was especially true at 1 DPN, although extremely large ribbons were seen at this time (Fig. 1). With time elapsed after the noise, the averaged ribbon size recovered and even become larger than that of the control at 1 MPN. This suggested that the newly produced ribbons were relatively larger. A significant effect of recovery time was seen in a two-way ANOVA. In contrast, the size changes in PSDs were not statistically significant.

**Figure 6:**
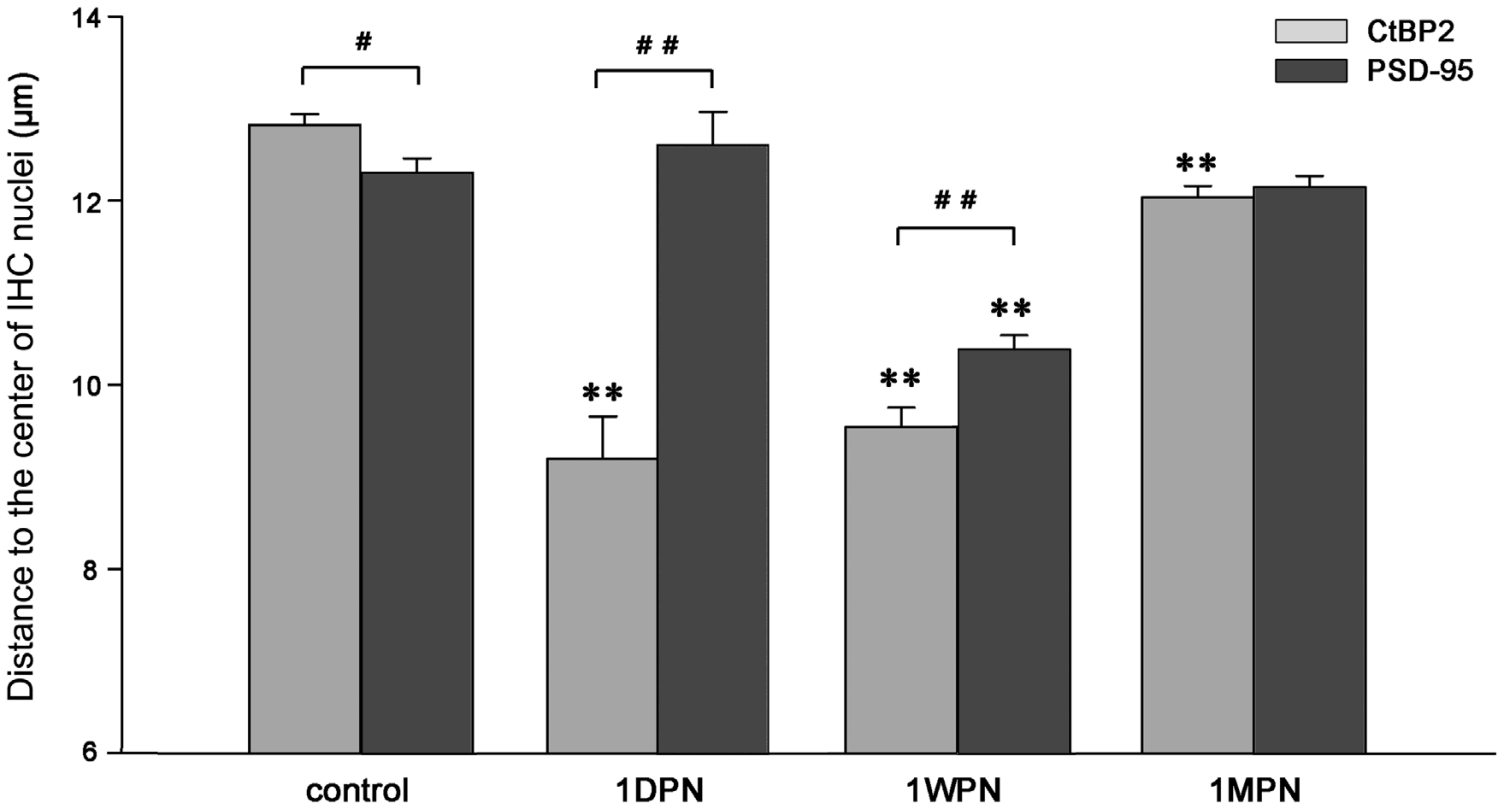
Location changes of ribbons and PSDs after noise exposure. A larger distance between the two structures and the IHC nuclei suggests a location towards the bottom of the IHCs. **: p<0.01 for both ribbons and PSDs, compared with the control. ##: p<0.01 for the comparison between ribbons and PSDs.

For the dislocation of ribbons and PSDs, we measured the distance between the center of the IHC nucleus and each PSD and each ribbon (Fig. 6). A large distance indicates a location towards the bottom of the IHCs. In each cochlea, distance data were collected from 10–20 IHCs in the 22–24-kHz region. Roughly 1,300, 350, 1000, and 1900 ribbons and PSDs were measured for the control, at 1 DPN, 1 WPN, and 1 MPN respectively. A two-way ANOVA was performed for the distance measured against the factor of noise (time) and targeted observation (ribbon vs. PSD). Significant differences were seen for the factors of time versus noise (F3 = 108.32, p<0.001) and the targeted synaptic component (F1 = 55.169, p<0.001). Because the distance was decreased significantly at 1 DPN for ribbons, but was slightly increased for PSDs, a significant difference between the two synaptic components was seen at this time (p<0.01, ## in Fig. 6), reflecting the separation between ribbons and PSDs because the ribbons (whether surviving or newly generated) were located towards the nuclei, while the PSDs remained at the bottom of the IHCs. At 1 WPN, however, the distance for ribbons had recovered (increased) slightly, while the distance between PSDs and the IHC nuclei was significantly reduced, compared with the control and 1 DPN values (p<0.01, ** in Fig. 6 for PSDs). Thus, the ribbons and PSDs seemed to attract each other and became closer. From 1 WPN to 1 MPN, both ribbons and PSDs appeared to be paired and moved back towards the bottom location, although the recovery of ribbon count was not complete.

### B. CAP to time-stress stimuli

**Figure 7:**
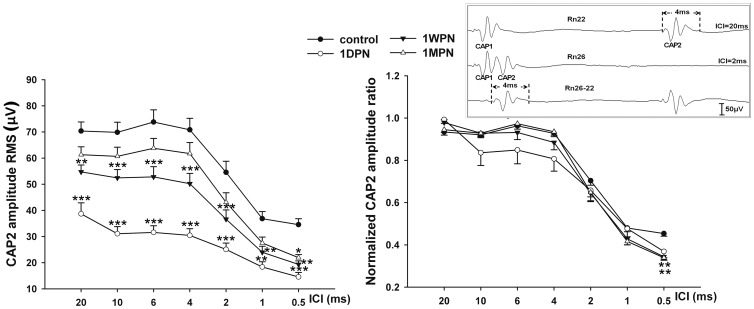
CAP amplitude changes as a function of inter-click interval. The CAP to the second click (CAP2) was measured in RMS. A: absolute amplitude, B: normalized amplitude ratio, using largest CAP2 as 100%. The level of clicks was 70 dB peSPL.

The impact of noise exposure on cochlear temporal coding was evaluated by the CAP responses to time-stress stimuli: paired clicks with varying ICIs at a moderate sound level (70 dB peSPL). The amplitude of CAP to the second click (CAP2) was plotted as a function of ICIs to address the CAP responses to time-stress with shortening ICIs. To avoid confusion in peak identification, the root-mean-square (RMS), instead of peak amplitude, was calculated in a 4-ms window, starting at the onset of the second click (see insert in Fig. 7). When the ICI was smaller than 4 ms, the response to the second click overlapped with that to the first click. In such cases, the response was subtracted with CAP recorded at 20 ms ICI to eliminate the response to the first click before calculating the RMS.

**Figure 8:**
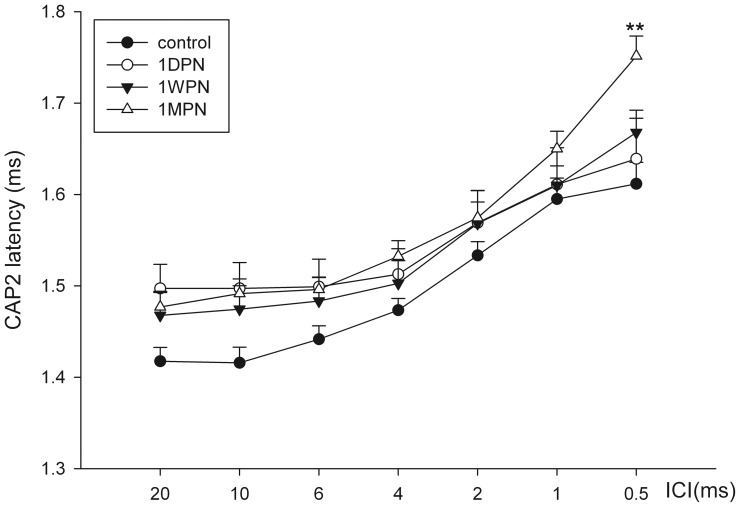
CAP2 latency as a function of ICIs. The response was tested at a level of 70

To understand how the CAP2 amplitude dropped with ICI, the absolute amplitude was converted to a ratio against the maximal CAP2, which was usually obtained at large ICIs (between 20 and 10 ms). Both absolute and normalized CAP2 amplitude declined as the ICI decreased. A more rapid decline in the CAP2 ratio with ICI is considered to indicate poorer temporal processing. An immediate and marked decrease on CAP2 amplitude was seen at 1 DPN (Fig. 7A), followed by an incomplete recovery within 1 month. A two-way ANOVA showed a significant effect of time after noise (*F_3,279_* = 96.7, p<0.001). A significant drop in absolute amplitude was seen across all the ICIs at 1 DPN and 1 WPN. However, similar analysis on normalized amplitude-ratio changes with ICIs (Fig. 7B) showed significance only in the shortest ICI (0.5 ms), at which a significantly bigger CAP2 ratio drop was seen at 1 WPN and 1 MPN (p<0.01), but not at 1 DPN. CAP2 latency was plotted as a function of ICIs in Figure 8 at 70 dB peSPL. An increase in the CAP2 latency was seen at the three time points after the noise. However, a significant difference was only seen at the shortest ICI and between the data at 1 MPN and the control.

### C. Changes in CAP amplitude

Corresponding to the synaptic damage and repair were the changes in CAP amplitude (Fig. 7). For CAP amplitude, we focused on the largest amplitude that was produced at a relatively high sound level. For CAP evoked by 80 dB peSPL clicks, the CAP amplitude was 371.28±7.19 in the control and 197.4±14.88, 239.01±12.59, and 272.29±19.58 at 1 DPN, 1 WPN, and 1 MPN respectively, a reduction of 46.8% at 1 DPN, which was reduced to 26.7% of the control at 1 MPN. Generally, the loss of CAP amplitude was larger than that of ribbon counts.

## Discussion

In the present study, we revisited how low-level/dose noise damages the ribbon synapses in the cochleae of guinea pigs. We focused on the dynamic changes in both synaptic ribbons and PSDs after the noise exposure and the functional consequences of the morphological changes. The major findings were as follows. (1) The numbers of both ribbons and PSDs were reduced shortly after the noise and recovered partially within 1 month, with a less than 10% residual loss that might be permanent. (2) The change in ribbon counts paralleled that of PSD counts, with more ribbon loss than PSD. (3) Shortly after the noise, many ribbons (residual or newly produced) were distributed towards the IHC nuclei while the surviving PSDs were remained at bottom position, resulting in many ribbons unpaired with PSDs. Later, the two structures appeared to attract each other, so the PSDs moved up to paired with down-moving ribbons. Eventually, the paired ribbons and PSDs moved down to the normal bottom position. This location change was accompanied by significant size changes in ribbons (first a decrease and then an increase) but not in PSD. (4) The CAP amplitude reduction was greater than was expected from the synapse numbers, suggesting functional damage to residual or repaired synapses. (5) In contrast to the recovery in ABR threshold and synapse number, temporal processing was found to worse towards the end of the observation period, at 1 MPN.

There were several interesting issues related to the repair process of the ribbon synapses observed in this study. The first was the dislocation of ribbons and PSDs. Some ribbons were located close to and even above the nuclei of the IHCs at 1 DPN (Fig. 1), resulting a significant reduction in the average distance between ribbons and the IHC nuclei (Fig. 6). At this time, however, the PSD location was not significantly changed (Fig. 6). This discrepancy correlated with the fact that many PSDs and ribbons were not paired in location at this time. It is unclear why the ribbons were dislocated towards the nuclei of the IHCs at that time. The ribbons observed at 1 DPN might have been the residual ribbons or newly generated after the damage. If the former is the case, it suggests that the ribbons located towards the bottom of the IHCs may be more sensitive to noise damage. We suggest that the dislocated ribbons are newly generated. This is supported by the organelles responsible for new protein synthesis (such as ribosomes and endoplasmic reticulum) all being located around the nucleus. In response to sound, some ribbons might have been broken down. The repair of the ribbons may require production of new ribbon protein. This should be evaluated in future experiments.

It may be argued that the dislocation of ribbons and PSDs are the artifacts due to the dislocation of IHC nuclei and/or the change in the length of IHCs. One possibility for such a dislocation is the large expansion of nerve terminals due to swelling caused by noise via glutamate. This swelling might have pushed the bottom of the IHC membrane up towards the nuclei. Such extremely swollen nerve terminals have been reported previously as the result of carboplatin insult [12] and the local application of kainic acid to the cochleae [13]–[15]. However, no such swelling was seen in our electronic microscopy observations of ribbon synapses after the noise exposure at 105 dB SPL in guinea pigs. Furthermore, the swelling, it occurred, should have pushed both PSDs and ribbons up and would not change the distances between those two types of puncta with the nuclei. In other words, the difference between the distances indicates the separation between the two types of puncta, whether or not the location of the nuclei is changed. Anyhow, the mismatch between ribbons and PSDs would be expected to be functionally significant, because those pre-synaptic ribbons cannot be involved in synaptic transmission. This could be one reason why the CAP amplitude reduction was larger than would be expected by the ribbon/PSD counts.

**Figure 9:**
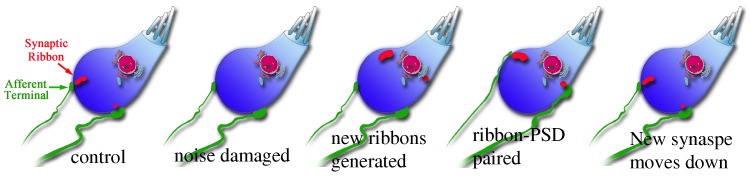
Schematic of the hypothesis of ribbon synapse plasticity after destruction by noise. We hypothesize that the noise exposure destroyed the ribbons, which were then reproduced via protein synthesis by organelles around the nuclei. The ribbons move laterally to cell membranes. Attraction between ribbons and PSDs causes downward and upward movements, respectively, of those structures. Eventually, the paired ribbon-PSDs move downwards.

Second, our data suggest significant synaptic plasticity after the noise and an interesting interaction between ribbons and PSDs. The newly generated ribbons appeared to be largely separated from the PSD at 1 DPN, with ribbons located closer to the nuclei of the IHCs. Between 1 DPN and 1 WPN, the PSDs seemed to be attracted by the ribbons, showing an upward movement. In this way, the two synaptic components would be better associated functionally. After that time (between 1 WPN and 1 MPN), both ribbons and PSDs returned back to the bottom location. Thus, we propose a process of synaptic plasticity, as shown in Figure 9. In this hypothesized process, we assume that the new ribbons are generated from the organelles responsible for the synthesis of new proteins. Because these organelles are located close to the nuclei of the IHC, the repaired ribbons are dislocated towards the nuclei. One thing we do not know is whether the new ribbons are free in the cytoplasm or anchored on the cell membrane, or even to other organelles. Later, we assume there is a strong attraction between ribbons and PSDs, which results in the upward movement of PSDs and downward movement of ribbons so that the two components move closer to each other, as indicated by our data. The attraction leads the ribbons and PSDs to be paired. After that, they move downwards, to the bottoms of the IHCs. At this time, we do not understand how this movement occurs. Exploring the underlying mechanisms may provide important information as to how to promote the repair process.

Before accepting this hypothesis, other possibilities must be considered. For example, the increased distance from 1 WPN to 1 MPN between both ribbon and PSDs with the nuclei of IHCs may not be due to the relocation of the synapses, but rather the changes in the length of IHCs or the distance between the nuclei and the base of IHCs. However, those two possibilities are unlikely to be true. We identified the lowest pair of Ribbon and PSD in each IHC to represent the far base of each IHC. The averaged value of the lowest puncta did not show significant difference between 1 WPN and 1 MPN.

Finally, the size of the ribbons and PSDs was first reduced. This was especially true for the ribbons. Interestingly, although the average ribbon size was reduced, there were also extremely large ribbons (Fig. 1, images at 1 DPN and 1 WPN). We suggest that the size change may be related to the damage and repair processes: the small ribbons may be the remnants of damaged ribbons, while the large ribbons are newly generated. Although we do not at present have evidence to support this, the phenomenon is worthy of further investigation. For example, we do not see such extremely large ribbons at 1 MPN. Does this suggest that the newly assembled ribbons shrink during maturation? At this moment, we do not know how accurate is the size determined by immunofluorescence staining. We are conducting an electron microscopy study to assess this.

In the present study, we used a PSD95 antibody to label the postsynaptic terminals instead of labeling glutamate receptors (GluRs). While the GluR is a good marker of the postsynaptic terminals of the IHC-SGN synapses in the sense of its high specificity, there is concern because of inducible changes in this receptor, by noise or glutamate: acoustic stimulation or application of glutamate agonists can significantly reduce the number of the receptors as a mechanism of self-protection [16]–[17]. Thus, noise-induced post-synaptic damage may be over-estimated if GluR is used as the indicator because the loss of GluR may not be accompanied by loss of terminals. In the present study, it was clear that the loss of PSD-labeled terminals was less than that of ribbons. This is the opposite to a previous study using GluR in which ribbon loss was found to be less than the loss of GluR-stained puncta [3].

The most striking functional change observed in this report is the great reduction and uncompleted recovery of CAP amplitude after the noise exposure (Figure 7). The amplitude reduction is generally larger than that of ribbon synapse loss. For example, the reduction in the click evoked CAP amplitude was more than 50% at 1 DPN, while the ribbon loss below 10 kHz (the frequency region covering the click spectrum) was roughly around 30%. This suggests that the survived ribbon synapses have functionally been impaired. Although the ABR threshold is fully recovered at 1 WPN, the CAP amplitude is only fully recovered even at 1 MPN.

The paired clicks stimuli exert time stress with decreasing ICI. This method has been used clinically and experimentally for the evaluation of temporal resolution [18]–[20]. Previously, we reported the deterioration of temporal processing using paired click-evoked ABRs [2]. At that time, we were unsure whether the deterioration was due to damage to ribbon synapses because the problem might occur in the cochlea or the brainstem. With the evaluation using CAP, we now can conclude that this deterioration originated in the cochlea, largely due to damage to the ribbon synapses. The normalized CAP-ICI function showed larger differences between the control and 1 MPN data at the shortest ICI (Fig. 7B). This is consistent with our previous report of a significant reduction in ABR2/ABR1 ratio at 1 ms ICI at 1 WPN and 1 MPN [2]. The larger reduction of CAP2 and ABR2 ratios at short ICIs after noise may be related to poorer recovery of the synaptic function of neurotransmitter release. Because the ABR2 and CAP2 largely overlapped with ABR1 and CAP2, the subtraction method may not have totally cancelled the overlap. In fact, whether the changes are really related to synaptic function must be tested further, ideally in single-unit recordings of 8^th^ nerve fibers.

The deficit in ribbon synapse function is likely due to a reduction in the rapid release of neurotransmitter. This is consistent with a recent report in which a mutation in Bassoon (a ribbon protein) was found to result in reduced neurotransmitter release to the second pulse in pairs (similar to the paired click paradigm used here) when the intervals between the two pulses were short [21]. For noise-induced damage, we assume that the repaired ribbon synapses are functionally disadvantaged, especially following signals that change rapidly. This is supported by the fact that the deficit was more significant at 1 MPN but not shortly after the noise exposure when more synapse damage was seen consistently in both CAP and ABR. It is also likely that the newly established synapses may not function well in temporal processing. Because they make a relatively larger contribution to the CAP during recovery towards 1 MPN, the temporal processing deficit was seen later. We are beginning a study to record responses from single auditory nerves to assess this. In a previous study in mice, the temporal processing ability was not reported [1]. In our own evaluation, we failed to find temporal processing ability changes in mice after a similar noise exposure that caused CAP amplitude reduction of more than 40% (Fig. S1). We think that this may have been due to the lack of synapse repair in this species.

It seems likely that the noise-induced damage to the IHC-SGN synapse can cause deterioration of temporal processing. Because the chance of being exposed to low-level noise is high in modern society, such damage can accumulate in the cochlea over a lifetime and thus may be one of the mechanisms, or even a major one, for the reduced temporal resolution observed in aging subjects [22]. Such deficits are considered major problems in signal processing with aging and the reason for difficulty in speech perception experienced by the elderly [23]–[28]. Protection against noise becomes more of a challenge because the noise that was previously considered to be safe is apparently not.

## Supporting Information

Figure S1 **The impact of noise exposure on CAP responses to paired clicks from CBA mice.** Similar noise exposure of 100 dB SPL for 2 h was used in this species as reported previously by Kujawa and Liberman. We found a permanent CAP amplitude reduction 4 weeks after the noise exposure. The amplitude was measured for CAP responses to the second clicks in the pair (CAP2) as a function of inter-click intervals (ICIs). This was designed to test the CAP2 response to time stress produced by reduced ICIs. A presents the functions for absolute amplitude, while B for amplitude ratio normalized against the largest CAP. B is specifically used to indicate the temporal processing ability of the cochlea. In the cochlea of guinea pigs, the ratio reduction with ICIs was significantly larger at the shortest ICI tested (0.5 ms). But this deficit was not seen in the cochlea of the mice. We hypothesize that the difference in CAP2 ratio-ICI functions between the species is due to the difference in the ability of ribbon synapse repair as explained in the manuscript.
(TIF)
