# Supplementary figures and images for: Ribbon Synapse Plasticity in the Cochleae of Guinea Pigs after Noise-Induced Silent Damage
Source: PLoS One. 2013 Dec 9;8(12):e81566. doi: 10.1371/journal.pone.0081566 (PMC3857186; doi:10.1371/journal.pone.0081566)

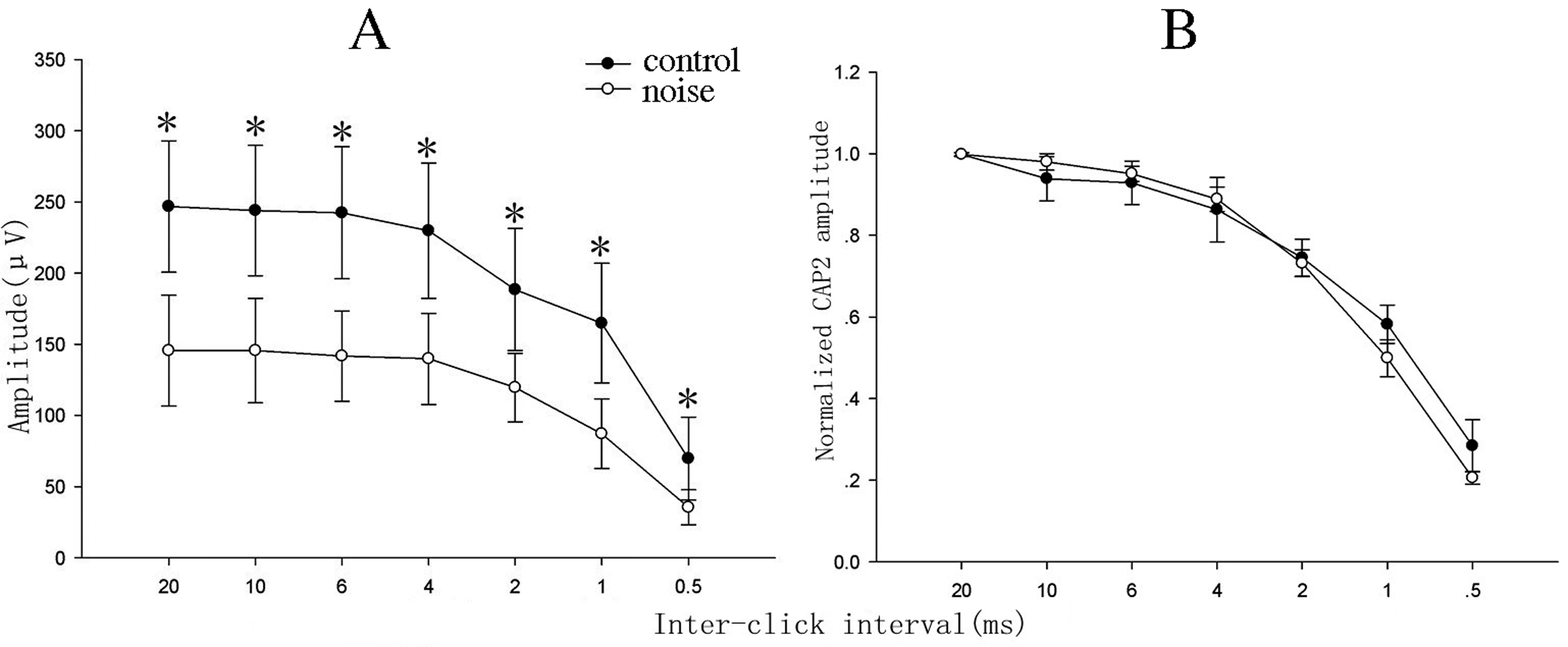

Supplement: Figure S1 — The impact of noise exposure on CAP responses to paired clicks from CBA mice. Similar noise exposure of 100 dB SPL for 2 h was used in this species as reported previously by Kujawa and Liberman. We found a permanent CAP amplitude reduction 4 weeks after the noise exposure. The amplitude was measured for CAP responses to the second clicks in the pair (CAP2) as a function of inter-click intervals (ICIs). This was designed to test the CAP2 response to time stress produced by reduced ICIs. A presents the functions for absolute amplitude, while B for amplitude ratio normalized against the largest CAP. B is specifically used to indicate the temporal processing ability of the cochlea. In the cochlea of guinea pigs, the ratio reduction with ICIs was significantly larger at the shortest ICI tested (0.5 ms). But this deficit was not seen in the cochlea of the mice. We hypothesize that the difference in CAP2 ratio-ICI functions between the species is due to the difference in the ability of ribbon synapse repair as explained in the manuscript. (TIF) [file pone.0081566.s001.tif]
